# The association between gestational weight gain z-score and stillbirth: a case-control study

**DOI:** 10.1186/s12884-019-2595-x

**Published:** 2019-11-29

**Authors:** Cassandra M. Pickens, Carol J. Hogue, Penelope P. Howards, Michael R. Kramer, Martina L. Badell, Donald J. Dudley, Robert M. Silver, Robert L. Goldenberg, Halit Pinar, George R. Saade, Michael W. Varner, Barbara J. Stoll

**Affiliations:** 1 Department of Epidemiology, Rollins School of Public Health, Emory University, 1518 Clifton Rd NE, Atlanta, GA 30322 USA; 2 Laney Graduate School, Emory University, 201 Dowman Dr, Atlanta, GA 30307 USA; 3 Department of Gynecology and Obstetrics, School of Medicine, Emory University, 1648 Pierce Dr NE, Atlanta, GA 30307 USA; 4 Department of Obstetrics and Gynecology, School of Medicine, University of Virginia, 1215 Lee St, Charlottesville, VA 22908 USA; 5 Department of Obstetrics and Gynecology, School of Medicine, University of Utah, 30 N 1900 E, Salt Lake City, UT 84132 USA; 6 Department of Obstetrics and Gynecology, Columbia University Medical Center, 630 W 168th St, New York, NY 10032 USA; 7 Department of Pathology and Laboratory Medicine, Brown University, 222 Richmond St, Providence, RI 02903 USA; 8 Department of Obstetrics and Gynecology, University of Texas Medical Branch at Galveston, 301 University Blvd, Galveston, TX 77555 USA; 9 Medical School, University of Texas Health Science Center at Houston, 7000 Fannin St #1200, Houston, TX 77030 USA

**Keywords:** Fetal death, Gestational weight gain, Obesity, Stillbirth

## Abstract

**Background:**

There is limited information on potentially modifiable risk factors for stillbirth, such as gestational weight gain (GWG). Our purpose was to explore the association between GWG and stillbirth using the GWG z−score.

**Methods:**

We analyzed 479 stillbirths and 1601 live births from the Stillbirth Collaborative Research Network case−control study. Women with triplets or monochorionic twins were excluded from analysis. We evaluated the association between GWG z−score (modeled as a restricted cubic spline with knots at the 5th, 50th, and 95th percentiles) and stillbirth using multivariable logistic regression with generalized estimating equations, adjusting for pre − pregnancy body mass index (BMI) and other confounders. In addition, we conducted analyses stratified by pre − pregnancy BMI category (normal weight, overweight, obese).

**Results:**

Mean GWG was 18.95 (SD 17.6) lb. among mothers of stillbirths and 30.89 (SD 13.3) lb. among mothers of live births; mean GWG z−score was − 0.39 (SD 1.5) among mothers of cases and − 0.17 (SD 0.9) among control mothers. In adjusted analyses, the odds of stillbirth were elevated for women with very low GWG z−scores (e.g., adjusted odds ratio (aOR) and 95% Confidence Interval (CI) for z−score − 1.5 SD versus 0 SD: 1.52 (1.30, 1.78); aOR (95% CI) for z−score − 2.5 SD versus 0 SD: 2.36 (1.74, 3.20)). Results differed slightly by pre − pregnancy BMI. The odds of stillbirth were slightly elevated among women with overweight BMI and GWG z−scores ≥1 SD (e.g., aOR (95% CI) for z−score of 1.5 SD versus 0 SD: 1.84 (0.97, 3.50)).

**Conclusions:**

GWG z−scores below − 1.5 SD are associated with increased odds of stillbirth.

## Background

Stillbirth (fetal death ≥20 weeks of gestation) occurs in 1 of every 168 U.S. pregnancies reaching 20 weeks of gestation [1]. The stillbirth rate among women with pre − pregnancy overweight and obesity is even higher [2]. Although the overall stillbirth rate decreased slightly in the past two decades, the gestation−specific rate for 20–27 week deliveries has not changed, and stillbirth is now more common than infant mortality in the U.S. [1]. There are limited data on potentially modifiable risk factors for stillbirth such as gestational weight gain (GWG) [3].

Gestational weight gain is associated with many risk factors for stillbirth independently of pre − pregnancy body mass index (BMI). High GWG is linked to maternal medical conditions, such as gestational diabetes [4] and hypertensive disorders [5, 6], and to altered fetal growth, such as macrosomia [7] and intrauterine growth restriction [8]. Inadequate GWG increases the risks of fetal growth restriction [7] and preterm birth [9].

Evidence regarding the association between stillbirth itself and maternal weight gain is limited. The 2009 Institute of Medicine (IOM) Committee to Reexamine Pregnancy Weight Guidelines requested research on gestational weight gain with stillbirth as a major endpoint [10]. However, many previous studies on GWG and stillbirth [11–14] have numerous limitations, including restricting to stillbirths ≥28 weeks [15].

Evaluating the relation between GWG and stillbirth is challenging because both variables are highly correlated with gestational age (GA) at delivery [16]. GWG varies over time and typically increases throughout pregnancy [17]. The vast majority of stillbirths are preterm, limiting the GWG timeframe [2]. A GWG z−score measure, which standardizes for GA, was recently proposed in order to account for this correlation [18–20]. Our objective was to evaluate the association between GWG z−score and the odds of stillbirth, while accounting for pre − pregnancy BMI.

## Methods

### Data source

The Stillbirth Collaborative Research Network (SCRN) Study was a multicenter case−control study conducted from 2006 to 2008 throughout Rhode Island and selected counties in Georgia, Massachusetts, Utah, and Texas. SCRN’s study methodology has been described in detail elsewhere [21]. Women with stillbirths (cases) and live births (controls) were enrolled at the time of delivery, with oversampling of women with preterm live births and non − Hispanic black women with live births at later gestational ages. Women had to be ≥13 years of age to participate and enrolled prior to hospital discharge. Data collection of consenting women included medical record abstraction, placental pathology, fetal autopsy, and a postpartum maternal interview [21]. Most interviews were completed face−to−face before hospital delivery discharge; a few interviews were completed by telephone or other method within 4 weeks of delivery. Sociodemographic information was derived from the maternal interview. Maternal height, pre − pregnancy weight, weight at last prenatal visit, and weight at delivery were abstracted from medical records. If maternal height or pre − pregnancy weight were unavailable in the medical record, women’s self−reported height and pre − pregnancy weight data were taken from the maternal postpartum interview (we used postpartum interview data on height and pre-pregnancy weight for only *n* = 1 woman in our analysis). Maternal pre-pregnancy body mass index (BMI) was categorized as underweight (BMI < 18.5 kg/m^2^), normal weight (BMI 18.5 − < 25.0 kg/m^2^), overweight (BMI 25.0 − < 30.0 kg/m^2^), class 1 obese (BMI 30.0 − < 35.0 kg/m^2^), and classes 2–3 obese (BMI ≥35.0 kg/m^2^).

Gestational age at delivery for both live births and stillbirths was determined via an algorithm that incorporated date and reliability of last menstrual period, ultrasound estimates of GA, and GA at study screening [22]. Gestational age at fetal death was determined via an algorithm based on fetal foot length and other measures [22]. Fourteen singleton stillbirths had an estimated GA at fetal death that was later than the estimated GA at delivery; GA at delivery was re-coded to GA at fetal death for these observations. Cause of fetal death was determined via a thorough review of autopsy reports, placental pathology, laboratory findings, and medical records [23]. Causes of fetal death were classified as probable causes, possible causes, or present conditions. Cause-of-death categories included placental abnormalities, obstetric conditions, fetal genetic/structural abnormalities, infection, umbilical cord abnormalities, hypertensive disorders, maternal medical conditions (excluding hypertension), and other causes [23]. Stillbirths could be classified as having multiple causes of death. Seventy-six percent of stillbirths in SCRN were assigned a probable or possible cause of death, and 31% of stillbirths had more than one possible or probable cause [23].

**Fig. 1:**
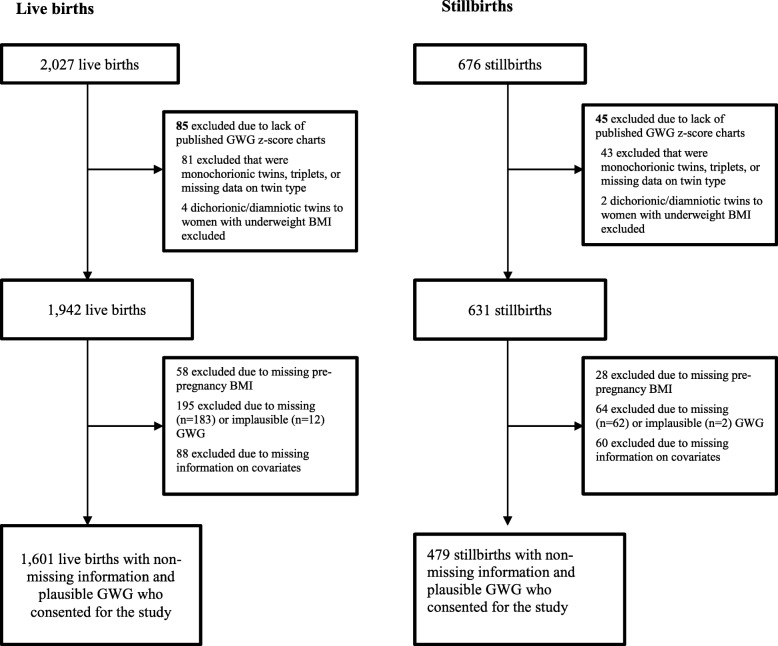
Study Exclusions by Case−Control Status. This figure depicts how many women were excluded at each successive step of sample selection. We excluded women with monochorionic/monoamniotic twin pregnancies, monochorionic/diamniotic twin pregnancies, triplet or higher−order gestations, missing or implausible GWG, and missing pre − pregnancy BMI or covariates

We excluded women with deliveries < 20 weeks, missing or implausible GWG (weight loss > 50 pounds or gain > 150 pounds), or missing pre − pregnancy BMI or covariates. Due to a lack of GWG z−score charts for certain groups, we excluded women with triplet gestations, monochorionic/monoamniotic twin pregnancies, and monochorionic/diamniotic twin pregnancies. We excluded women who had dichorionic/diamniotic twin pregnancies and underweight BMI but included women who had dichorionic/diamniotic twin pregnancies and normal weight, overweight, or obese BMI (Fig. 1).

### Exposure measure

Total GWG was defined as maternal weight at delivery minus pre − pregnancy weight. We calculated GWG z−scores using published charts that were developed from a follow−up study of healthy Pittsburgh women who delivered term live births [18–20]. Gestational weight gain (in kg) was transformed to a z−score using the published formula $\frac{\ln \left( GWG+c\right)- mean\ \left(\ln (GWG)\right)}{standard\ deviation\left(\ln (GWG)\right)}$ [18–20]. The mean and standard deviation depended on pre − pregnancy BMI category and GA at delivery, while c was a constant that transformed ln(GWG) to a positive value (means, standard deviations, and the constant were taken from published references [18–20]). GWG z−scores can be calculated for either ongoing or completed pregnancies and require only three measurements: pre − pregnancy weight, weight at delivery (or weight at the GA in question), and GA at delivery (or at the time in question) [18–20]. Among women with singleton pregnancies, published GWG z−score charts end at 40 weeks for women with normal weight and at 41 weeks for women with overweight or obesity; among women with dichorionic twin pregnancies, published charts end at 39 weeks for women with normal weight and at 38 weeks for women with overweight/obesity. In our main analyses, gestational ages above these cutoffs were rounded down to the last gestational week available in the chart (e.g., 40 weeks in singleton pregnancies to women with normal weight).

### Statistical analyses

We conducted data analysis in SAS (Cary, NC). To examine associations between GWG z−score and stillbirth, we used univariable and multivariable logistic regression models with generalized estimating equations that accounted for correlation between multiple gestations. We chose an independent correlation structure after initial models showed a negligible difference between exchangeable and independent correlation structures. All analyses were weighted to account for SCRN’s sampling design and individuals’ probabilities of participating and completing all parts of the data collection process [21]. Models were adjusted for maternal age at delivery, maternal race and ethnicity, study site (Rhode Island/Massachusetts, Georgia, Galveston (Texas), San Antonio (Texas), Utah), maternal education, marital status/cohabitating, health insurance type, trimester prenatal care began, family income in the last 12 months, WIC enrollment, smoking or alcohol consumption during the 3 months prior to pregnancy, lifetime drug use, pregnancy history, history of hypertension, history of preexisting diabetes, history of thyroid disorder, and pre-pregnancy BMI category. Potential confounders, including maternal race and ethnicity, were selected a priori using directed acyclic graphs based on evidence of their associations with GWG and stillbirth [2].

We modeled GWG z−score as a restricted cubic spline with three knots corresponding to the 5th, 50th, and 95th percentiles (percentiles were calculated among live births only using SCRN analysis weights in SAS) [24]. We then calculated odds ratio contrasts of interest by comparing selected GWG z−scores (ranging from − 2.5 to 2.5, in intervals of 0.5) to a referent GWG z−score of 0. In addition, we used GWG z−score models stratified by pre − pregnancy BMI category (normal weight, overweight, obese (BMI ≥30.0 kg/m^2^)) [25, 26]. We were unable to analyze women with underweight BMI separately due to inadequate sample size. To use a restricted cubic spline in models stratified by pre − pregnancy BMI category, we re − calculated the 5th, 50th, and 95th percentiles of GWG z−score separately in each BMI category among live birth controls using SCRN analysis weights. Models among women with BMI ≥30.0 kg/m^2^ were adjusted for obesity class.

### Sensitivity analyses

To examine how different assumptions affected results, we conducted various sensitivity analyses. These included: 1) using separate models for women with class 1 obesity versus classes 2–3 obesity; 2) restricting to women with stillbirths estimated to have died ≤1 day before delivery in order to limit the potential for reverse causality (i.e., to limit the risk of the fetal death itself influencing total GWG), and comparing these results to a model restricted to women with stillbirths estimated to have died > 1 day before delivery [22]; 3) restricting to stillbirths estimated to have been alive at their last prenatal visit [22], as well as re-calculating GWG z−scores for these stillbirths using weight and GA at last prenatal visit, in order to limit potential for reverse causality (a small number of stillbirths without prenatal care were excluded from this sensitivity analysis); 4) excluding mummified stillbirths (grade IV or higher maceration among fragmented fetuses and grade V or higher maceration among intact fetuses) because these stillbirths may have a significant discrepancy between fetal weight at death and delivery; 5) analyzing stillbirths by timing of delivery (< 28 weeks, ≥28 weeks, < 37 weeks, and ≥ 37 weeks) in order to explore potential etiologic differences; 6) restricting to women with non − anomalous antepartum stillbirths or non − anomalous live births; 7) analyzing intrapartum stillbirths separately due to their differing pathophysiology [23]; 8) excluding stillbirths with causes of death related to fetal genetic, structural, or karyotypic abnormalities or maternal/fetal hematologic conditions [27] because fetal weight may be driven more by congenital abnormalities than by maternal nutritional status in these pregnancies [28]; 9) excluding stillbirths that had an estimated GA at fetal death < 20 weeks [22], despite having a GA at delivery ≥20 weeks; 10) excluding women with a GA at delivery that exceeded the limit on the GWG z−score charts [18–20]; 11) using weight at last prenatal visit as an estimate of weight at delivery for women who were missing delivery weight (last prenatal visit is typically a few days before delivery [29] for term pregnancies); 12) controlling for weight and height squared as separate variables because of concern about introducing bias with the use of ratio measures [30]; and 13) analyzing stillbirths by three cause-of-death categories [23]. For this final sensitivity analysis, we first restricted to stillbirths with a probable cause of death, possible cause of death, or present condition related to placental abnormalities (e.g., uteroplacental insufficiency, maternal vascular disorders) [23]. Second, we restricted to stillbirths with a probable cause of death, possible cause of death, or present condition related to maternal medical conditions, excluding hypertension (e.g., diabetes, antiphospholipid syndrome, thyroid disorder) [23]. Third, we restricted to stillbirths with a probable cause of death, possible cause of death, or present condition related to obstetric conditions (e.g., placental abruption; complications of multiple gestations; the combination of preterm labor, preterm premature rupture of membranes, and cervical insufficiency) [23].

### Ethics and consent

This study was reviewed and approved by the Institutional Review Boards of each of the participating sites (Brown University, Emory University, University of Texas Health Science Center at San Antonio, University of Texas Medical Branch at Galveston, University of Utah) and by the data coordinating center (RTI International). Written informed consent was obtained from participants or from their legal guardians (if participants were minors).

## Results

Of 1991 eligible live births and 652 eligible stillbirths, we excluded 390 live births and 173 stillbirths for reasons outlined above, leaving 1601 live births and 479 stillbirths to consented participants (Fig. 1). Mothers of stillbirths were more likely than mothers of live births to be non − Hispanic black, < 20 or ≥ 35 years old, non − married/non − cohabitating, and to have a previous stillbirth (Table 1). Mothers of stillbirths were also more likely to have preexisting hypertension, preexisting diabetes, and an above normal pre − pregnancy BMI.

**Table 1:** Frequencies of maternal characteristics by case−control status

	Stillbirths (*n* = 479)		Live births (*n* = 1601)	
	n	Weighted %	n	Weighted %
Plurality				
Singleton	470	98.2	1544	96.7
Twin (dichorionic/diamniotic)	9	1.8	57	3.3
Maternal age at delivery, years				
< 20	61	13.2	193	10.5
20–34	341	69.6	1194	75.1
35–39	59	13.0	186	12.5
≥40	18	4.2	28	1.9
Maternal race and ethnicity				
White, non − Hispanic	184	36.0	596	45.3
Black, non − Hispanic	82	18.4	268	10.5
Hispanic	176	38.5	620	35.7
Other	37	7.1	117	8.5
Study site				
State of Rhode Island and Bristol County, Massachusetts	86	16.8	311	25.4
DeKalb County, Georgia	73	15.4	288	12.6
Galveston County and Brazoria County, Texas	49	9.3	134	9.0
Bexar County, Texas	143	35.3	506	30.2
Salt Lake County, Utah	128	23.2	362	22.8
Maternal education, grade				
0–11 (none/primary/some secondary)	110	23.1	330	18.2
≥12 (completed secondary)	369	77.0	1271	81.8
Marital status/cohabitating				
Not married or cohabitating	110	24.0	273	14.1
Cohabitating or married	369	76.0	1328	85.9
Health insurance/method of payment				
Received any public/private assistance or did not have insurance	275	58.9	923	52.2
VA/commercial health insurance/HMO^a^	204	41.2	678	47.9
Trimester prenatal care began				
First	325	67.1	1077	72.2
Second	110	23.0	407	22.2
Third or no prenatal care	44	10.0	117	5.7
Family income in the last 12 months				
Received public/private assistance	206	44.2	751	42.8
Only personal income	273	55.8	850	57.2
WIC enrollment^b^				
Yes	161	34.4	620	35.7
No	318	65.6	981	64.3
Used alcohol or smoked during the 3 months prior to pregnancy				
Yes	220	45.7	696	47.1
No	259	54.3	905	52.9
Lifetime drug use				
Ever	161	33.1	470	30.5
Never	318	66.9	1131	69.5
Pregnancy history				
Primiparous	224	46.3	564	34.5
Multiparous with no previous stillbirth	223	46.7	996	64.1
Multiparous with previous stillbirth	32	7.1	41	1.4
Pre − pregnancy Body Mass Index				
Underweight (BMI < 18.5 kg/m^2^)	18	3.5	51	3.1
Normal weight (BMI 18.5 − < 25.0 kg/m^2^)	185	38.3	760	49.8
Overweight (BMI 25.0 − < 30.0 kg/m^2^)	119	24.9	393	23.3
Class 1 obese (BMI 30.0 − < 35.0 kg/m^2^)	79	16.9	211	12.7
Classes 2–3 obese (BMI ≥35.0 kg/m^2^)	78	16.4	186	11.1
Clinical history of hypertension				
Yes	43	9.4	113	6.6
No	436	90.6	1488	93.4
Clinical history of diabetes				
Yes	26	5.6	37	1.7
No	453	94.4	1564	98.3
Clinical history of thyroid disorder				
Yes	19	3.7	59	3.4
No	460	96.3	1542	96.6
Gestational age at delivery, weeks				
20–23	135	28.9	95	0.6
24–27	86	17.8	87	0.6
28–31	65	12.5	78	1.0
32–36	98	21.3	129	8.6
≥37	95	19.6	1212	89.2

^a^*VA* Veterans Affairs, *HMO* Health Maintenance Organization

^b^Special Supplemental Nutrition Program for Women, Infants, and Children

The average gestational age at delivery was 38.6 weeks for live births and 29.6 weeks for stillbirths, and the average estimated GA at fetal death was 28.6 weeks (Table 2). The mean difference between GA at delivery and estimated GA at fetal death (as estimated by the SCRN timing-of-death algorithm [22]) among stillbirths was 1.02 weeks, while the median difference was 0.29 weeks (SD: 1.7 weeks). Women with stillbirths had lower mean values of total GWG and GWG z−score than women with live births (Table 2). Mothers of stillbirths had a mean GWG of 18.95 lb. (SD 17.6 lb) and mean GWG z−score of − 0.39 (SD 1.5), while mothers of live births had a mean GWG of 30.89 lb. (SD 13.3 lb) and mean GWG z−score of − 0.17 (SD 0.9). Mean total GWG was inversely associated with pre − pregnancy BMI category, while the highest mean GWG z−scores were in women with BMI < 18.5 or ≥ 35.0 kg/m^2^. Mean GWG z−scores were negative for control mothers with normal weight, overweight, and class 1 obesity (Table 2).

**Table 2:** Distributions of gestational age, total GWG, and GWG Z−scores by case−control status^a^

	Stillbirths (*n* = 479)			Live births (*n* = 1601)		
Gestational age at delivery, weeks	Mean		SD	Mean		SD
	29.60		6.6	38.64		2.1
Estimated gestational age at fetal death, weeks (stillbirths only)^b^	Mean		SD			
	28.58		7.0			
Total GWG, lb	Mean		SD	Mean		SD
Total sample (All BMI Categories)	18.95		17.6	30.89		13.3
Underweight (BMI < 18.5 kg/m^2^)	25.23		19.9	35.12		10.3
Normal weight (BMI 18.5 − < 25.0 kg/m^2^)	21.65		15.2	33.90		11.7
Overweight (BMI 25.0 − < 30.0 kg/m^2^)	22.13		19.5	30.75		12.8
Class 1 obese (BMI 30.0 − < 35.0 kg/m^2^)	15.96		17.5	26.24		14.0
Classes 2–3 obese (BMI ≥35.0 kg/m^2^)	9.55		15.7	21.90		16.2
GWG Z−scores	Mean	Median	SD	Mean	Median	SD
Total sample (All BMI Categories)	−0.39	−0.26	1.5	−0.17	−0.10	0.9
Underweight (BMI < 18.5 kg/m^2^)	−0.23	−0.80	1.8	0.14	0.26	0.9
Normal weight (BMI 18.5 − < 25.0 kg/m^2^)	− 0.63	− 0.40	1.8	− 0.20	− 0.17	1.0
Overweight (BMI 25.0 − < 30.0 kg/m^2^)	− 0.26	−0.30	1.3	−0.26	−0.17	0.8
Class 1 obese (BMI 30.0 − < 35.0 kg/m^2^)	− 0.31	− 0.17	1.2	−0.17	−0.19	0.9
Classes 2–3 obese (BMI ≥35.0 kg/m^2^)	−0.12	−0.01	1.1	0.09	0.24	0.7

^a^Means, medians, and standard deviations are weighted, but sample sizes are unweighted

^b^Gestational age at fetal death was determined via an algorithm based on fetal foot length and other measures [22]

In unadjusted analyses, GWG z−scores at or below − 0.5 SD were associated with increased odds of stillbirth; associations were stronger for z−scores below − 1.5 SD (e.g., crude odds ratio (cOR) and 95% Confidence Interval (CI) for − 1.5 vs. 0 SD: 1.46 (1.26, 1.70); cOR (95% CI) for − 2.5 SD: 2.25 (1.67, 3.02); Additional file 1). In addition, GWG z−scores at or above 1.5 SD were associated with slightly increased odds of stillbirth (cOR (95% CI) for GWG z−scores of 1.5 SD: 1.35 (1.03, 1.76); cOR (95% CI) for 2.5 SD: 1.79 (1.11, 2.87)).

**Fig. 2:**
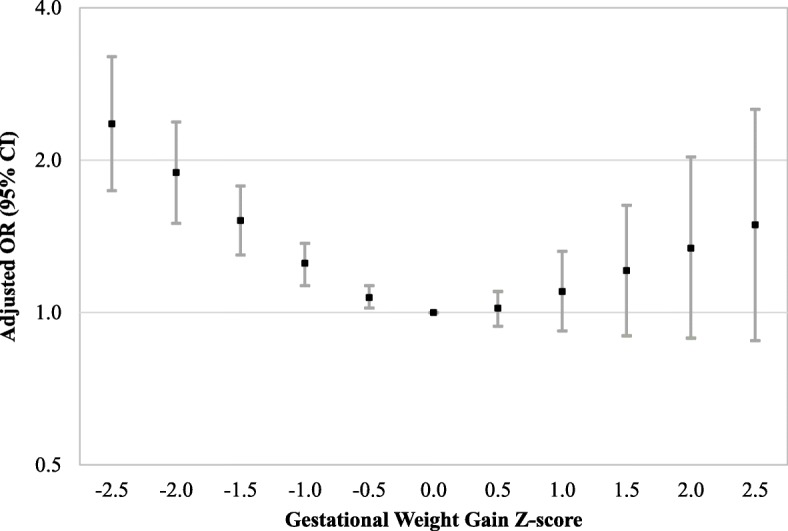
Association of Gestational Weight Gain Z−scores with Stillbirth. This figure displays adjusted odds ratios for the association between GWG Z−scores and stillbirth. Selected GWG z−scores were compared to a referent z−score of 0. Models were adjusted for maternal age at delivery, maternal race and ethnicity, study site, maternal education, marital status/cohabitating, health insurance type, trimester prenatal care began, family income in the last 12 months, WIC enrollment, smoking or alcohol consumption during the 3 months prior to pregnancy, lifetime drug use, pregnancy history, history of hypertension, history of preexisting diabetes, and history of thyroid disorder

Associations for low GWG z−scores (≤ − 0.5 SD) were similar after adjusting for covariates (Fig. 2; Additional file 1). In adjusted analyses, women with GWG z−scores of − 1.5 SD had a 1.52 (95% CI 1.30, 1.78) times increased odds of stillbirth, while women with GWG z−scores of − 2.5 SD had a 2.36 (1.74, 3.20) times increased odds of stillbirth. Confidence intervals for high GWG z−scores (≥1.5 SD) overlapped the null in adjusted analyses (e.g., adjusted odds ratio (aOR) for z−score of 2 SD: 1.34 (95% CI 0.89, 2.03); Additional file 1).

**Fig. 3:**
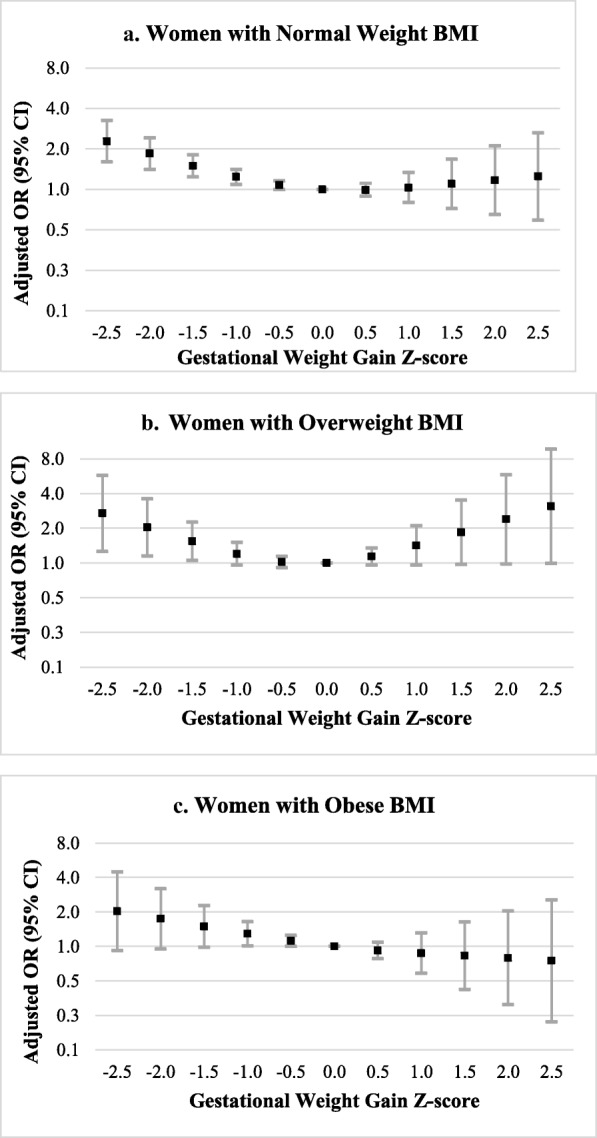
Adjusted Odds Ratios for Gestational Weight Gain Z−scores and Stillbirth by Pre − pregnancy BMI Category. This figure displays adjusted odds ratios for the association between GWG Z−scores and stillbirth, stratified by pre − pregnancy BMI category (normal weight (**a**), overweight (**b**), obese (**c**)). Selected GWG z−scores were compared to a referent z−score of 0. Models were adjusted for maternal age at delivery, maternal race and ethnicity, study site, maternal education, marital status/cohabitating, health insurance type, trimester prenatal care began, family income in the last 12 months, WIC enrollment, smoking or alcohol consumption during the 3 months prior to pregnancy, lifetime drug use, pregnancy history, history of hypertension, history of preexisting diabetes, and history of thyroid disorder. The model among women with obesity was also adjusted for obesity class (1, 2, 3) (**c**)

Associations between stillbirth and GWG z−scores < 0 were similar by pre – pregnancy BMI category (normal weight, overweight, or obese; see Fig. 3 and Additional file 2). For instance, the odds of stillbirth were increased by approximately 50% in women with GWG z−scores of − 1.5 SD, regardless of pre – pregnancy BMI category. However, precision was reduced compared to main analyses. Associations at very low z−scores (− 2.5 SD) were stronger in women with overweight BMI. Additionally, among women with overweight BMI, the odds of stillbirth were elevated at high GWG z−scores (e.g., aOR (95% CI) for GWG z−score of 1.5 SD: 1.84 (0.97, 3.50); for GWG z−score of 2.5 SD: 3.10 (0.99, 9.68)). Among women with obese BMI, adjusted ORs decreased from 2.02 to 0.75 as GWG z−scores increased from − 2.5 to 2.5 SD, but most 95% CIs overlapped the null.

**Fig. 4:**
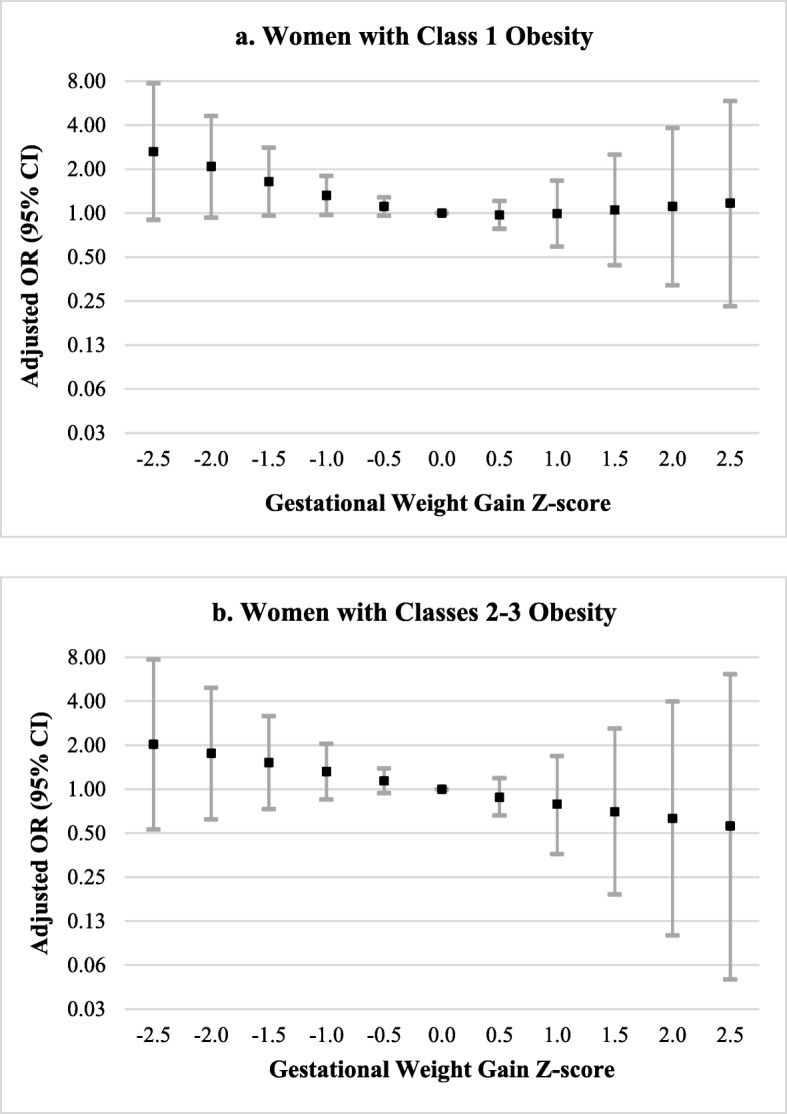
Association of GWG Z−scores with Stillbirth among Women with Obesity. This figure displays adjusted odds ratios for the association between GWG Z−scores and stillbirth, stratified by obesity severity. Adjusted odds ratios among women with class 1 obesity are in **a**; adjusted odds ratios among women with classes 2–3 obesity are in **b**. Selected GWG z−scores were compared to a referent z−score of 0. Models were adjusted for maternal age at delivery, maternal race and ethnicity, study site, maternal education, marital status/cohabitating, health insurance type, trimester prenatal care began, family income in the last 12 months, WIC enrollment, smoking or alcohol consumption during the 3 months prior to pregnancy, lifetime drug use, pregnancy history, history of hypertension, history of preexisting diabetes, and history of thyroid disorder. Models among women with classes 2–3 obesity were also adjusted for obesity class (2, 3)

Sample sizes of each sensitivity analysis are presented in Additional file 3. Figure 4 and Additional file 4 display aORs for the association between GWG z−scores and stillbirth, stratified by obesity severity (class 1 obesity versus classes 2 and 3 obesity). The mean GWG z−scores among mothers of live births differed by obesity severity (class 1 obese: − 0.17 (SD 0.9); classes 2–3 obese: 0.09 (SD 0.7)). Similar to main analyses, the odds of stillbirth were elevated for women with obesity and GWG z−scores ≤ − 0.5 SD (e.g., aOR for z−score of − 1.5 SD among women with class 1 obesity: 1.64 (95% CI 0.96, 2.80); among women with classes 2–3 obesity: 1.52 (95% CI 0.73, 3.17)). However, associations were imprecise. GWG z−scores > 0 SD were not associated with stillbirth among women with class 1 obesity. Among women with classes 2–3 obesity, the odds of stillbirth were slightly reduced for GWG z−scores ≥1 SD, but confidence intervals overlapped the null (e.g., aOR for GWG z−score of 2 SD: 0.63 (95% CI 0.10, 3.99)).

Results from additional sensitivity analyses are shown in Additional files 5, 6, and 7. Results from most sensitivity analyses were similar to the main models. For instance, results were similar to main analyses after restricting to stillbirths estimated to have died ≤1 day or > 1 day before delivery, particularly for z-scores between − 2.5 and 1 SD. Point estimates for GWG z−scores ≥1.5 SD were slightly further from the null after restricting to stillbirths estimated to have died ≤1 day before delivery, while aORs for GWG z−scores ≥1.5 SD were slightly closer to the null when restricting to stillbirths estimated to have died > 1 day before delivery, though 95% CIs overlapped (Additional file 5). Associations between low GWG z−scores and stillbirth were somewhat attenuated when we restricted to stillbirths that were alive at their last prenatal visit and re-calculated GWG z−scores for these stillbirths using weight and GA at last prenatal visit (e.g., aOR for − 2 SD: 1.42 (95% CI 1.00, 2.02); Additional file 5). In contrast, associations between high GWG z−scores and stillbirth were stronger in this sensitivity analysis (e.g., aOR for 2 SD: 2.62 (95% CI 1.70, 4.04); Additional file 5). Associations from a sensitivity analysis excluding macerated stillbirths, as well as a sensitivity analysis excluding stillbirths with causes of death related to congenital anomalies or hematologic conditions, were similar to main analyses (Additional files 5 and 6). In models with intrapartum stillbirths only, aORs decreased with increasing GWG z−scores (aOR for GWG z−score of − 2.0: 1.50 (95% CI 0.87, 2.59); aOR for GWG z−score of 2.0: 0.62 (95% CI 0.22, 1.76)); however, confidence intervals were wide (Additional file 5).

Adjusted odds ratios for the association between GWG z−scores < 0 and stillbirth were similar in stillbirths delivered < 28 weeks as in stillbirths delivered ≥28 weeks (Additional file 5). In stillbirths ≥28 weeks only, the odds of stillbirth were elevated among women with very high GWG z−scores (e.g., aOR for 2.5 SD: 1.91 (95% CI 1.00, 3.64)). Associations among stillbirths < 37 weeks were similar to main analyses, but GWG z−score was not associated with the odds of stillbirth ≥37 weeks (Additional file 5). Findings for low GWG z−scores (i.e., z−scores < 0) were similar when we analyzed stillbirths by cause of death (i.e., when we restricted to stillbirths with placental abnormalities, restricted to stillbirths with maternal medical complications of pregnancy (excluding hypertension), and restricted to stillbirths with obstetric conditions; Additional file 7). However, findings for high GWG z−scores and stillbirth were slightly different by cause-of-death group. In analyses restricted to stillbirths with obstetric complications of pregnancy, very high GWG z−scores were associated with increased odds of stillbirth (e.g., aOR for z−score of 2 SD: 2.36 (95% CI 1.28, 4.35)). In contrast, associations between GWG z−scores > 0 and stillbirth were closer to the null in analyses restricted to stillbirths with maternal medical conditions excluding hypertension. Additional sensitivity analyses yielded comparable results to main analyses.

## Discussion

Our analysis suggests that a low GWG z−score is associated with slightly increased odds of stillbirth, as compared to a referent GWG z−score of 0. Associations were strongest at very low GWG z−scores: for instance, the odds of stillbirth were elevated up to 2.36 times for GWG z−scores ≤ − 2.5 SD. However, associations were weaker near z−scores of 0, i.e., in GWG z−score ranges where the majority of pregnant women fall. High GWG z−score was not associated with the odds of stillbirth in the overall sample. In women with overweight BMI, the odds of stillbirth were increased at GWG z−scores ≥1 SD.

One pathway through which low GWG could influence stillbirth is through preterm labor [9, 23]. Low GWG is a risk factor for preterm delivery [9]. If a fetus cannot tolerate preterm labor, intrapartum stillbirth could occur [23]. Alternatively, the association between low GWG z−score and stillbirth may be driven by intrauterine growth restriction [7]. Beginning in the second trimester, low GWG z−score may be an indicator of poor fetal weight gain. However, we cannot determine whether poor fetal growth caused stillbirth or, alternatively, whether fetuses at higher risk of stillbirth simply stopped growing as a result of congenital or placental/intrauterine complications. However, it is unlikely that the associations between low GWG z−scores and stillbirth in our study were due to stillbirths with birth defects or hematologic conditions, as a sensitivity analysis excluding stillbirths with these conditions produced similar results to main analyses.

A “net” z−score (total GWG minus fetal, placental, and amniotic fluid weight) would allow a more thorough evaluation of the impact of fetal versus maternal weight gain, but there are no published “net GWG” percentiles from the referent populations we used. Our dataset also lacked information on weight of the placenta and amniotic fluid, which typically weigh 2–3 pounds combined [10], as well as on plasma volume.

High GWG z−score was not associated with the odds of stillbirth in our overall sample. Associations between high GWG z−score and risk factors for stillbirth, such as preeclampsia, may be weak in our study sample. In addition, although excess GWG has been linked to many adverse maternal outcomes [4–6, 31], a GWG level that is harmful for the mother may not always be harmful for the fetus [10].

In stratified analyses, our findings differed slightly by pre – pregnancy BMI category. Our sensitivity analyses also suggested that the association between high GWG z−score and stillbirth may differ between women with class 1 obesity versus classes 2–3 obesity. However, precision was limited in sensitivity analyses. We also lacked an adequate sample size to further separate women with class 2 obesity from women with class 3 obesity. Future research using larger sample sizes of women with classes 2–3 obesity could be informative.

Gestational weight gain z−score was not associated with the odds of stillbirth ≥37 weeks. In SCRN, stillbirths ≥37 weeks were less likely than stillbirths < 37 weeks to have probable or possible causes of death related to obstetric complications or hypertensive disorders [23]. Our analyses by cause of death are subject to certain limitations. We were unable to analyze all biologically relevant causes of fetal death, such as hypertensive disorders, due to inadequate sample size. We also lacked the sample size to restrict to stillbirths with only *probable* causes of death related to obstetric complications, maternal medical conditions, or placental disease; instead, we combined those with probable causes, possible causes, and present conditions [23].

Evidence on the association between GWG and stillbirth is sparse [11–14, 32]. Johansson et al. recently evaluated the association between both early GWG (< 22 weeks) and total GWG and the risk of stillbirth ≥22 weeks [32] using GWG z−scores derived from a Swedish reference chart [33]. The authors found no association between early GWG or total GWG and stillbirth at most z−score ranges [32]. Point estimates at extreme z−scores suggested a possible association between very low GWG z−score (e.g., < − 2 SD) and reduced risk of stillbirth, as well as very high z−score (e.g., > 2 SD) and increased risk of stillbirth, but 95% CIs overlapped the null [32]. Johansson et al.’s findings of no association at GWG z−score ranges where the majority of pregnant women fall (i.e., between − 1.5 and 1.5 SD) are comparable to our findings. The four remaining previous studies of stillbirth had various limitations [11–14]. All four were restricted to stillbirths ≥28 weeks (in concordance with the World Health Organization’s definition [15]); however, stillbirths at 20–27 weeks constitute half of stillbirths in the U.S. [1]. Furthermore, two studies excluded intrapartum stillbirths [11, 13], two did not account for GA in adjusted analyses [12, 14], and one excluded women with gestational diabetes or hypertensive disorders [11], which are plausible consequences of GWG [10]. Despite these methodological differences, our finding of an overall null association between high GWG z−score and stillbirth was in concordance with four of five previous studies [11–13, 32]. Although our stratified analyses suggested a possible association between high GWG z−score and stillbirth among women with pre-pregnancy overweight or classes 2–3 obesity, future studies with larger sample sizes are necessary to make firm conclusions. Our observation showing increased odds of stillbirth at low GWG z−scores is consistent with trends from three previous reports [11, 13, 14], although confidence intervals from some prior studies overlapped the null [11, 13].

Analyzing the association between GWG and stillbirth is challenging given the relatively low incidence of stillbirth, need for high−quality data, and importance of properly accounting for GA. In our case−control study of stillbirth, we standardized for GA using the GWG z−score, which is straightforward to calculate [18–20]. In addition to using GWG z−scores, Johansson et al. used an incidence density sampling approach to match stillbirths and live births on GA of last GWG measurement in a large prospective Swedish birth cohort of over 160,000 deliveries with detailed GWG data [32]. Survival analysis is another possible approach to evaluating the association between GWG and stillbirth in a cohort study while accounting for GA. However, prospective studies are impractical in many cases and require extremely large sample sizes for rare outcomes such as stillbirth.

Our analytic methods have limitations. We used GWG at delivery. Ideally, gestational weight gain should be measured shortly before fetal death occurs. However, bias due to time of measurement is likely limited in our study. Results from sensitivity analyses excluding macerated stillbirths, who may have notable discrepancies between fetal weight at death and delivery, were similar to main analyses. Furthermore, the mean interval between estimated GA at fetal death (as estimated by the SCRN timing-of-death algorithm [22]) and delivery was only 1.02 weeks (median: 0.29 weeks), and findings from sensitivity analyses restricted to stillbirths estimated to have died ≤1 day (versus > 1 day) before delivery were largely similar to main analyses. There may be some bias for stillbirths with a longer time interval between fetal death and delivery. Point estimates also shifted somewhat when we restricted to stillbirths who were alive at their last prenatal visit and re-calculated GWG z−scores for these stillbirths using weight and GA at last prenatal visit. However, overall trends were similar to main analyses. Of note, the preceding sensitivity analysis did not consider GWG occurring between the last prenatal visit and fetal death (mean time interval between last prenatal visit and fetal death was 2.5 (SD 3.2) weeks for stillbirths in the analysis). An additional limitation is that our analysis assumes that GWG z−score at delivery (which is used in the analysis) is equivalent to GWG z−score earlier in pregnancy. An ideal study design would compare GWG z-scores for stillbirths and live births at the same GA.

The GWG z−scores in our study, as derived from Hutcheon et al.’s cohort [18–20], may not be entirely independent of gestational duration. A recent study found that GWG z−scores remained slightly correlated with GA when Hutcheon et al.’s z−score formulas were applied in the Consortium for Safe Labor study population [34]. This issue may arise if the relation between GWG and GA differs between Hutcheon et al.’s cohort and the study population of interest [34]. The GWG distribution in our study population does differ slightly from Hutcheon et al.’s cohort, as evidenced by the non – zero median GWG z−scores among SCRN control mothers. Our study population also differed in certain other ways from that of Hutcheon et al.; e.g., mothers in SCRN were slightly younger (mean maternal age in our dataset was 27.5 years, versus 29 years [18–20]) and were more likely to be Hispanic. If GWG z−scores in our study remain correlated with GA, results for low GWG z−score and stillbirth could be biased up and away from the null. Still, Hutcheon et al.’s z−score charts have many strengths that are relevant to our study [18–20]. Hutcheon et el.’s charts [18–20] are available for all pre-pregnancy BMI categories (unlike certain other z−score charts [35]) and twins (unlike all other charts [33, 35, 36]), are derived from a U.S. population, and are based on a follow-up study with a large number of measured weights during pregnancy (in contrast, a recent published GWG z−score chart included cohorts with self-reported weight measurements during pregnancy [36]). Hutcheon et al.’s charts have recently been applied to the general U.S. population in studies of GWG and preterm birth [37, 38].

Thirteen percent of eligible live births and 14.7% of eligible stillbirths in our study had missing information on GWG or pre – pregnancy BMI. However, in sensitivity analyses, we used maternal weight at last prenatal visit as an estimate of delivery weight for more than 55% of observations missing delivery weight, and results were unchanged. We did not control for gestational diabetes, although a gestational diabetes diagnosis could plausibly influence women’s GWG in late pregnancy. This was a purposeful decision, as gestational diabetes may be an intermediate between GWG and stillbirth. Another potential weakness is an inability to control for all potential confounders due to limited sample size or lack of information on these factors (e.g., physical activity [39]). Lastly, our results may not be generalizable to certain women with multiple gestations (e.g., women with monochorionic twin pregnancies, triplets, or higher−order births).

Our study has many strengths. To our knowledge, it is the first analysis of GWG and stillbirth to evaluate cause-specific stillbirth, to include twins, and to examine differences by obesity severity. Furthermore, it is only the second analysis to utilize the GWG z−score and to include stillbirths at 20–27 weeks [32]. We conducted extensive sensitivity analyses evaluating how the timing and cause of fetal death, maceration level, and numerous other factors influenced results. Furthermore, SCRN sampled women from geographically and demographically diverse catchment areas and did not restrict to academic or tertiary care hospitals [21]. SCRN’s source population is also well−enumerated, with analysis weights that account for study design and probability of participation [21]. Finally, SCRN’s comprehensive data collection process provided detailed information on maternal covariates as well as timing and cause of fetal death [21].

Investigators have recently advocated for the widespread use of the GWG z−score in research (and ultimately, clinical) settings across diverse populations [18, 19, 34]. The GWG z−score’s predictive ability for stillbirth is likely limited due to the relatively modest aORs observed in our study. However, the z−score may prove useful for stillbirth in combination with other clinical measures, such as estimated fetal size. Results from our analyses suggest that very low GWG z−score may be a marker of poor fetal health.

## Conclusions

Gaining at or below − 1.5 SD of GWG z−score may increase the odds of stillbirth.

## Supplementary information

**Additional file 1.** Unadjusted and Adjusted Odds Ratios for GWG Z−scores and Stillbirth among Women of All Pre − pregnancy BMI Categories. This table displays the unadjusted and adjusted odds ratios for the association between GWG z−scores and stillbirth among women of all pre − pregnancy BMI categories. Selected GWG z−scores were compared to a referent z−score of 0. Adjusted models involved control for maternal sociodemographic, behavioral, and pregnancy characteristics.

**Additional file 2.** Unadjusted and Adjusted Odds Ratios for GWG Z−scores and Stillbirth by Pre-pregnancy BMI Category. This table displays the unadjusted and adjusted odds ratios for the association between GWG z−scores and stillbirth by pre-pregnancy BMI category (normal weight, overweight, obese). Selected GWG z−scores were compared to a referent z−score of 0. Adjusted models involved control for maternal sociodemographic, behavioral, and pregnancy characteristics.

**Additional file 3.** Sample Sizes for Sensitivity Analyses. This table shows the sample size for each sensitivity analysis.

**Additional file 4.** Adjusted Odds Ratios for GWG Z−scores and Stillbirth by Obesity Class. This table displays adjusted odds ratios for the association between GWG z−scores and stillbirth, stratified by obesity class (class 1 obesity (BMI 30.0 − < 35.0 kg/m^2^) and classes 2–3 obesity (BMI ≥35.0 kg/m^2^)). Selected GWG z−scores were compared to a referent z−score of 0. Adjusted models involved control for maternal sociodemographic, behavioral, and pregnancy characteristics.

**Additional file 5.** Adjusted Odds Ratios for GWG Z−scores and Stillbirth from Key Sensitivity Analyses. This table contains adjusted odds ratios for the association between GWG z−scores and stillbirth for key sensitivity analyses, including restricting to stillbirths estimated to have died ≤1 day before delivery and analyzing stillbirths by gestational age at delivery (< 28 vs. ≥28 weeks, < 37 vs. ≥37 weeks). Selected GWG z−scores were compared to a referent z−score of 0. Adjusted models involved control for maternal sociodemographic, behavioral, and pregnancy characteristics.

**Additional file 6.** Adjusted Odds Ratios for GWG Z−scores and Stillbirth from Additional Sensitivity Analyses. This table contains adjusted odds ratios for the association between GWG z−scores and stillbirth for various additional sensitivity analyses (e.g., excluding women with a GA at delivery that exceeded the limit on the GWG z−score charts; using weight at last prenatal visit for women missing delivery weight). Selected GWG z−scores were compared to a referent z−score of 0. Adjusted models involved control for maternal sociodemographic, behavioral, and pregnancy characteristics.

**Additional file 7.** Adjusted Odds Ratios for GWG Z−scores and Stillbirth by Cause-of-Death Groupings. This table contains adjusted odds ratios for the association between GWG z−scores and stillbirth by cause-of-death groupings [23]. The first sensitivity analysis was restricted to stillbirths with a probable cause of death, possible cause of death, or present condition related to placental abnormalities. The second was restricted to stillbirths with a probable cause of death, possible cause of death, or present condition related to maternal medical conditions excluding hypertension [23]. The third was restricted to stillbirths with a probable cause of death, possible cause of death, or present condition related to obstetric conditions [23]. Selected GWG z−scores were compared to a referent z−score of 0. Adjusted models involved control for maternal sociodemographic, behavioral, and pregnancy characteristics.

## Data Availability

The datasets generated and/or analyzed during the current study are publicly available in the National Institute of Child Health and Human Development (NICHD) Data and Specimen Hub (DASH) repository [https://dash.nichd.nih.gov/]. Also see https://www.nichd.nih.gov/research/supported/Pages/scrn.aspx for more information on the study background.

## Abbreviations

aOR: Adjusted odds ratio
BMI: Body mass index
CI: Confidence interval
COD: Cause of death
cOR: Crude odds ratio
GA: Gestational age
GWG: Gestational weight gain
HMO: Health Maintenance Organization
SCRN: Stillbirth Collaborative Research Network
SD: Standard deviation
VA: Veterans Affairs
WIC: Special Supplemental Nutrition Program for Women, Infants, and Children
