# Supplementary material for: The association between gestational weight gain z-score and stillbirth: a case-control study
Source: BMC Pregnancy Childbirth. 2019 Nov 29;19:451. doi: 10.1186/s12884-019-2595-x (PMC6883690; doi:10.1186/s12884-019-2595-x)
Supplement: Supplementary file 1 — Additional file 1. Unadjusted and Adjusted Odds Ratios for GWG Z−scores and Stillbirth among Women of All Pre − pregnancy BMI Categories. This table displays the unadjusted and adjusted odds ratios for the association between GWG z−scores and stillbirth among women of all pre − pregnancy BMI categories. Selected GWG z−scores were compared to a referent z−score of 0. Adjusted models involved control for maternal sociodemographic, behavioral, and pregnancy characteristics. [file 12884_2019_2595_MOESM1_ESM.docx]

**Additional File 1. Unadjusted and Adjusted Odds Ratios for GWG Z-scores and Stillbirth among Women of All Pre-pregnancy BMI Categories**

| **GWG Z-score^a^** | **Unadjusted OR [95% CI]** | **Adjusted OR [95% CI]^b^** |
| --- | --- | --- |
| **-2.5** | 2.25 (1.67, 3.02) | 2.36 (1.74, 3.20) |
| **-2.0** | 1.81 (1.45, 2.26) | 1.89 (1.50, 2.38) |
| **-1.5** | 1.46 (1.26, 1.70) | 1.52 (1.30, 1.78) |
| **-1.0** | 1.21 (1.10, 1.32) | 1.25 (1.13, 1.37) |
| **-0.5** | 1.05 (1.01, 1.10) | 1.07 (1.02, 1.13) |
| **0** | 1.00 (1.00, 1.00) | 1.00 (1.00, 1.00) |
| **0.5** | 1.05 (0.98, 1.12) | 1.02 (0.94, 1.10) |
| **1.0** | 1.17 (1.00, 1.38) | 1.10 (0.92, 1.32) |
| **1.5** | 1.35 (1.03, 1.76) | 1.21 (0.90, 1.63) |
| **2.0** | 1.55 (1.07, 2.25) | 1.34 (0.89, 2.03) |
| **2.5** | 1.79 (1.11, 2.87) | 1.49 (0.88, 2.52) |

**Description:** This table displays the unadjusted and adjusted odds ratios for the association between GWG z-score and stillbirth.

^a^Selected GWG z-scores were compared to a referent z-score of 0. Among women with singleton pregnancies, GWG z-scores of -2.5, -2.0, -1.5, -1.0, -0.5, 0, 0.5, 1.0, 1.5, 2.0, and 2.5 correspond to the following 40-week total GWG: in women with pre-pregnancy underweight, 11.8 lb, 15.1 lb, 18.9 lb, 23.2 lb, 28.1 lb, 33.6 lb, 40.0 lb, 47.2 lb, 55.4 lb, 64.7 lb, and 75.4 lb, respectively; in women with pre-pregnancy normal weight, 11.7 lb, 15.5 lb, 19.8 lb, 24.7 lb, 30.1 lb, 36.2 lb, 43.0 lb, 50.6 lb, 59.2 lb, 68.8 lb, and 79.5 lb, respectively; in women with pre-pregnancy overweight, 3.1 lb, 8.0 lb, 13.5 lb, 19.8 lb, 26.9 lb, 34.9 lb, 44.0 lb, 54.3 lb, 65.9 lb, 79.1 lb, and 94.0 lb, respectively; in women with pre-pregnancy class 1 obesity, -5.0 lb, 0.1 lb, 5.9 lb, 12.4 lb, 19.9 lb, 28.4 lb, 38.1 lb, 49.2 lb, 61.8 lb, 76.2 lb, and 92.5 lb, respectively; in women with pre-pregnancy class 2 obesity, -13.8 lb, -8.8 lb, -2.9 lb, 4.1 lb, 12.3 lb, 21.9 lb, 33.2 lb, 46.6 lb, 62.3 lb, 80.7 lb, and 102.4 lb, respectively; and among women with pre-pregnancy class 3 obesity, -22.7 lb, -18.0 lb, -12.2 lb, -5.0 lb, 4.0 lb, 15.1 lb, 28.9 lb, 46.0 lb, 67.1 lb, 93.4 lb, and 125.9 lb, respectively. Among women with dichorionic/diamniotic twin pregnancies, GWG z-scores of -2.5, -2.0, -1.5, -1.0, -0.5, 0, 0.5, 1.0, 1.5, 2.0, and 2.5 correspond to the following 38-week total GWG: among women with pre-pregnancy normal weight, 17.5 lb, 21.9 lb, 26.9 lb, 32.5 lb, 38.7 lb, 45.9 lb, 53.9 lb, 62.9 lb, 73.1 lb, 84.6 lb, and 97.6 lb, respectively; in women with pre-pregnancy overweight, 8.0 lb, 12.9 lb, 18.6 lb, 25.2 lb, 33.0 lb, 42.0 lb, 52.6 lb, 64.9 lb, 79.3 lb, 96.1 lb, and 115.8 lb, respectively; in women with pre-pregnancy obesity, -2.1 lb, 2.7 lb, 8.4 lb, 15.4 lb, 23.8 lb, 33.9 lb, 46.2 lb, 60.9 lb, 78.8 lb, 100.3 lb, and 126.3 lb, respectively.

^b^The model was adjusted for maternal age at delivery, maternal race and ethnicity, study site, maternal education, marital status/cohabitating, health insurance type, trimester prenatal care began, family income in the last 12 months, WIC enrollment, smoking or alcohol consumption during the 3 months prior to pregnancy, lifetime drug use, pregnancy history, history of hypertension, history of preexisting diabetes, and history of thyroid disorder.
