# Supplementary material for: The association between gestational weight gain z-score and stillbirth: a case-control study
Source: BMC Pregnancy Childbirth. 2019 Nov 29;19:451. doi: 10.1186/s12884-019-2595-x (PMC6883690; doi:10.1186/s12884-019-2595-x)
Supplement: Supplementary file 2 — Additional file 2. Unadjusted and Adjusted Odds Ratios for GWG Z−scores and Stillbirth by Pre-pregnancy BMI Category. This table displays the unadjusted and adjusted odds ratios for the association between GWG z−scores and stillbirth by pre-pregnancy BMI category (normal weight, overweight, obese). Selected GWG z−scores were compared to a referent z−score of 0. Adjusted models involved control for maternal sociodemographic, behavioral, and pregnancy characteristics. [file 12884_2019_2595_MOESM2_ESM.docx]

**Additional File 2. Unadjusted and Adjusted Odds Ratios for GWG Z-scores and Stillbirth by Pre-pregnancy BMI Category**

|  | **Pre-pregnancy normal weight** | | **Pre-pregnancy Overweight** | | **Pre-pregnancy Obesity** | |
| --- | --- | --- | --- | --- | --- | --- |
| **GWG Z-score^a^** | **Unadjusted OR (95% CI)** | **Adjusted OR (95% CI)^b^** | **Unadjusted OR (95% CI)** | **Adjusted OR (95% CI)^b^** | **Unadjusted OR (95% CI)** | **Adjusted OR (95% CI)^b^** |
| **-2.5** | 2.55 (1.73, 3.78) | 2.28 (1.60, 3.26) | 2.15 (1.10, 4.22) | 2.70 (1.26, 5.76) | 1.99 (1.00, 3.97) | 2.02 (0.92, 4.48) |
| **-2.0** | 1.99 (1.49, 2.67) | 1.85 (1.41, 2.42) | 1.71 (1.03, 2.83) | 2.03 (1.15, 3.59) | 1.70 (1.00, 2.87) | 1.74 (0.95, 3.18) |
| **-1.5** | 1.56 (1.28, 1.91) | 1.50 (1.24, 1.81) | 1.36 (0.97, 1.91) | 1.54 (1.05, 2.26) | 1.44 (1.01, 2.07) | 1.49 (0.98, 2.27) |
| **-1.0** | 1.26 (1.11, 1.44) | 1.24 (1.09, 1.41) | 1.12 (0.92, 1.36) | 1.20 (0.96, 1.51) | 1.24 (1.00, 1.53) | 1.29 (1.01, 1.64) |
| **-0.5** | 1.08 (1.00, 1.16) | 1.08 (1.00, 1.16) | 0.99 (0.90, 1.09) | 1.02 (0.91, 1.14) | 1.08 (0.98, 1.19) | 1.12 (1.00, 1.25) |
| **0** | 1.00 (1.00, 1.00) | 1.00 (1.00, 1.00) | 1.00 (1.00, 1.00) | 1.00 (1.00, 1.00) | 1.00 (1.00, 1.00) | 1.00 (1.00, 1.00) |
| **0.5** | 1.01 (0.91, 1.13) | 0.99 (0.89, 1.11) | 1.16 (1.01, 1.33) | 1.14 (0.96, 1.35) | 0.99 (0.85, 1.15) | 0.92 (0.78, 1.09) |
| **1.0** | 1.09 (0.85, 1.39) | 1.03 (0.80, 1.34) | 1.46 (1.07, 2.00) | 1.42 (0.96, 2.11) | 1.03 (0.71, 1.48) | 0.87 (0.58, 1.31) |
| **1.5** | 1.20 (0.80, 1.80) | 1.10 (0.72, 1.68) | 1.90 (1.14, 3.18) | 1.84 (0.97, 3.50) | 1.08 (0.59, 2.00) | 0.83 (0.42, 1.63) |
| **2.0** | 1.32 (0.75, 2.33) | 1.17 (0.65, 2.11) | 2.49 (1.22, 5.07) | 2.39 (0.98, 5.82) | 1.14 (0.48, 2.69) | 0.79 (0.31, 2.03) |
| **2.5** | 1.46 (0.71, 3.02) | 1.25 (0.59, 2.65) | 3.24 (1.30, 8.07) | 3.10 (0.99, 9.68) | 1.20 (0.40, 3.63) | 0.75 (0.22, 2.54) |

**Description:** This table displays the unadjusted and adjusted odds ratios for the association between GWG z-score and stillbirth by pre-pregnancy BMI category (normal weight, overweight, obese).

^a^Selected GWG z-scores were compared to a referent z-score of 0. Among women with singleton pregnancies, GWG z-scores of -2.5, -2.0, -1.5, -1.0, -0.5, 0, 0.5, 1.0, 1.5, 2.0, and 2.5 correspond to the following 40-week total GWG: in women with pre-pregnancy normal weight, 11.7 lb, 15.5 lb, 19.8 lb, 24.7 lb, 30.1 lb, 36.2 lb, 43.0 lb, 50.6 lb, 59.2 lb, 68.8 lb, and 79.5 lb, respectively; in women with pre-pregnancy overweight, 3.1 lb, 8.0 lb, 13.5 lb, 19.8 lb, 26.9 lb, 34.9 lb, 44.0 lb, 54.3 lb, 65.9 lb, 79.1 lb, and 94.0 lb, respectively; in women with pre-pregnancy class 1 obesity, -5.0 lb, 0.1 lb, 5.9 lb, 12.4 lb, 19.9 lb, 28.4 lb, 38.1 lb, 49.2 lb, 61.8 lb, 76.2 lb, and 92.5 lb, respectively; in women with pre-pregnancy class 2 obesity, -13.8 lb, -8.8 lb, -2.9 lb, 4.1 lb, 12.3 lb, 21.9 lb, 33.2 lb, 46.6 lb, 62.3 lb, 80.7 lb, and 102.4 lb, respectively; and among women with pre-pregnancy class 3 obesity, -22.7 lb, -18.0 lb, -12.2 lb, -5.0 lb, 4.0 lb, 15.1 lb, 28.9 lb, 46.0 lb, 67.1 lb, 93.4 lb, and 125.9 lb, respectively. Among women with dichorionic/diamniotic twin pregnancies, GWG z-scores of -2.5, -2.0, -1.5, -1.0, -0.5, 0, 0.5, 1.0, 1.5, 2.0, and 2.5 correspond to the following 38-week total GWG: among women with pre-pregnancy normal weight, 17.5 lb, 21.9 lb, 26.9 lb, 32.5 lb, 38.7 lb, 45.9 lb, 53.9 lb, 62.9 lb, 73.1 lb, 84.6 lb, and 97.6 lb, respectively; in women with pre-pregnancy overweight, 8.0 lb, 12.9 lb, 18.6 lb, 25.2 lb, 33.0 lb, 42.0 lb, 52.6 lb, 64.9 lb, 79.3 lb, 96.1 lb, and 115.8 lb, respectively; in women with pre-pregnancy obesity, -2.1 lb, 2.7 lb, 8.4 lb, 15.4 lb, 23.8 lb, 33.9 lb, 46.2 lb, 60.9 lb, 78.8 lb, 100.3 lb, and 126.3 lb, respectively.

^b^Adjusted for maternal age at delivery, maternal race and ethnicity, study site, maternal education, marital status/cohabitating, health insurance type, trimester prenatal care began, family income in the last 12 months, WIC enrollment, smoking or alcohol consumption during the 3 months prior to pregnancy, lifetime drug use, pregnancy history, history of hypertension, history of preexisting diabetes, and history of thyroid disorder. Adjusted models in obese women were adjusted for obesity class (1, 2, 3).
