# Supplementary material for: The association between gestational weight gain z-score and stillbirth: a case-control study
Source: BMC Pregnancy Childbirth. 2019 Nov 29;19:451. doi: 10.1186/s12884-019-2595-x (PMC6883690; doi:10.1186/s12884-019-2595-x)
Supplement: Supplementary file 3 — Additional file 3. Sample Sizes for Sensitivity Analyses. This table shows the sample size for each sensitivity analysis. [file 12884_2019_2595_MOESM3_ESM.docx]

**Additional File 3. Sample Sizes for Sensitivity Analyses**

| **Description of sensitivity analysis** | **Stillbirths (n)** | **Live births (n)** |
| --- | --- | --- |
| Restricting to women with stillbirths estimated to have died ≤ 1 day before delivery | 178 | 1,599 |
| Restricting to women with stillbirths estimated to have died >1 day before delivery | 299 | 1,598 |
| Restricting to stillbirths who were estimated to have been alive at their last prenatal visit, and calculating GWG z-score for stillbirths using weight and gestational age at last prenatal visit | 258 | 1,601 |
| Restricting to stillbirths <28 weeks | 221 | 1,601 |
| Restricting to stillbirths ≥28 weeks | 258 | 1,601 |
| Restricting to stillbirths <37 weeks | 384 | 1,601 |
| Restricting to stillbirths ≥37 weeks | 95 | 1,601 |
| Restricting to stillbirths with placental disease | 203 | 1,601 |
| Restricting to stillbirths with maternal medical complications of pregnancy, excluding hypertension | 129 | 1,601 |
| Restricting to stillbirths with obstetric complications | 143 | 1,601 |
|  |  |  |
| Restricting to women with non-anomalous antepartum stillbirths or non-anomalous live births | 356 | 1,548 |
| Restricting to women with intrapartum stillbirths or livebirths | 68 | 1,598 |
| Excluding pregnancies with probable causes of death related to fetal genetic, structural, or karyotypic abnormalities or maternal/fetal hematologic conditions | 318 | 1,598 |
| Excluding pregnancies with possible or probable causes of death related to fetal genetic, structural, or karyotypic abnormalities or maternal/fetal hematologic conditions | 306 | 1,598 |
| Excluding pregnancies with present, possible, or probable causes of death related to fetal genetic, structural, or karyotypic abnormalities or maternal/fetal hematologic conditions | 261 | 1,598 |
| Excluding pregnancies with mummified/macerated stillbirths | 439 | 1,600 |
| Excluding stillbirths with estimated GA at death <20 weeks | 444 | 1,601 |
| Excluding women with a GA at delivery that exceeded the limit on the GWG z-score charts | 468 | 1,411 |
| Restricting to women with class 1 obesity | 79 | 211 |
| Restricting to women with classes 2-3 obesity | 78 | 186 |
| Using weight at last prenatal visit as estimate of delivery weight for women missing delivery weight | 513 | 1,710 |
| Adjusting for weight and height^2^ as separate variables | 479 | 1,601 |
