# Supplementary material for: The association between gestational weight gain z-score and stillbirth: a case-control study
Source: BMC Pregnancy Childbirth. 2019 Nov 29;19:451. doi: 10.1186/s12884-019-2595-x (PMC6883690; doi:10.1186/s12884-019-2595-x)
Supplement: Supplementary file 4 — Additional file 4. Adjusted Odds Ratios for GWG Z−scores and Stillbirth by Obesity Class. This table displays adjusted odds ratios for the association between GWG z−scores and stillbirth, stratified by obesity class (class 1 obesity (BMI 30.0 − < 35.0 kg/m2) and classes 2–3 obesity (BMI ≥35.0 kg/m2)). Selected GWG z−scores were compared to a referent z−score of 0. Adjusted models involved control for maternal sociodemographic, behavioral, and pregnancy characteristics. [file 12884_2019_2595_MOESM4_ESM.docx]

|  | **Women with Class 1 Obesity** | | **Women with Classes 2−3 Obesity** | |
| --- | --- | --- | --- | --- |
| **GWG Z-score^a^** | **Crude OR (95% CI)** | **Adjusted OR**  **(95% CI)^b^** | **Crude OR**  **(95% CI)** | **Adjusted OR**  **(95% CI)^b^** |
| **−2.5** | 1.93 (0.75, 5.00) | 2.63 (0.90, 7.74) | 2.16 (0.80, 5.79) | 2.03 (0.53, 7.72) |
| **−2.0** | 1.62 (0.80, 3.29) | 2.08 (0.93, 4.63) | 1.83 (0.85, 3.94) | 1.76 (0.62, 4.94) |
| **−1.5** | 1.36 (0.85, 2.18) | 1.64 (0.96, 2.80) | 1.56 (0.91, 2.69) | 1.52 (0.73, 3.17) |
| **−1.0** | 1.16 (0.89, 1.53) | 1.32 (0.97, 1.80) | 1.33 (0.96, 1.84) | 1.32 (0.85, 2.05) |
| **−0.5** | 1.04 (0.91, 1.19) | 1.11 (0.96, 1.28) | 1.14 (0.98, 1.32) | 1.14 (0.94, 1.39) |
| **0** | 1.00 (1.00, 1.00) | 1.00 (1.00, 1.00) | 1.00 (1.00, 1.00) | 1.00 (1.00, 1.00) |
| **0.5** | 1.04 (0.85, 1.27) | 0.97 (0.78, 1.21) | 0.91 (0.74, 1.13) | 0.88 (0.66, 1.19) |
| **1.0** | 1.14 (0.70, 1.84) | 0.99 (0.59, 1.67) | 0.87 (0.50, 1.49) | 0.79 (0.36, 1.69) |
| **1.5** | 1.28 (0.57, 2.86) | 1.05 (0.44, 2.51) | 0.83 (0.33, 2.08) | 0.70 (0.19, 2.60) |
| **2.0** | 1.45 (0.47, 4.52) | 1.11 (0.32, 3.83) | 0.79 (0.22, 2.92) | 0.63 (0.10, 3.99) |
| **2.5** | 1.65 (0.38, 7.16) | 1.17 (0.23, 5.84) | 0.76 (0.14, 4.10) | 0.56 (0.05, 6.13) |

**Additional File 4. Adjusted Odds Ratios for GWG Z−scores and Stillbirth by Obesity Class**

^a^Selected GWG z−scores were compared to a referent z−score of 0. Among women with singleton pregnancies, GWG z−scores of −2.5, −2.0, −1.5, −1.0, −0.5, 0, 0.5, 1.0, 1.5, 2.0, and 2.5 correspond to the following 40−week total GWG: in women with pre−pregnancy class 1 obesity, −5.0 lb, 0.1 lb, 5.9 lb, 12.4 lb, 19.9 lb, 28.4 lb, 38.1 lb, 49.2 lb, 61.8 lb, 76.2 lb, and 92.5 lb, respectively; in women with pre−pregnancy class 2 obesity, −13.8 lb, −8.8 lb, −2.9 lb, 4.1 lb, 12.3 lb, 21.9 lb, 33.2 lb, 46.6 lb, 62.3 lb, 80.7 lb, and 102.4 lb, respectively; and among women with pre−pregnancy class 3 obesity, −22.7 lb, −18.0 lb, −12.2 lb, −5.0 lb, 4.0 lb, 15.1 lb, 28.9 lb, 46.0 lb, 67.1 lb, 93.4 lb, and 125.9 lb, respectively. Among women with dichorionic/diamniotic twin pregnancies and pre−pregnancy obesity, GWG z−scores of −2.5, −2.0, −1.5, −1.0, −0.5, 0, 0.5, 1.0, 1.5, 2.0, and 2.5 correspond to a 38−week total GWG of −2.1 lb, 2.7 lb, 8.4 lb, 15.4 lb, 23.8 lb, 33.9 lb, 46.2 lb, 60.9 lb, 78.8 lb, 100.3 lb, and 126.3 lb, respectively.

^b^Adjusted for maternal age at delivery, maternal race and ethnicity, study site, maternal education, marital status/cohabitating, health insurance type, trimester prenatal care began, family income in the last 12 months, WIC enrollment, smoking or alcohol consumption during the 3 months prior to pregnancy, lifetime drug use, pregnancy history, history of hypertension, history of preexisting diabetes, and history of thyroid disorder. The adjusted model in classes 2-3 obese women was also adjusted for obesity class (2, 3).
