# Supplementary material for: The association between gestational weight gain z-score and stillbirth: a case-control study
Source: BMC Pregnancy Childbirth. 2019 Nov 29;19:451. doi: 10.1186/s12884-019-2595-x (PMC6883690; doi:10.1186/s12884-019-2595-x)
Supplement: Supplementary file 5 — Additional file 5. Adjusted Odds Ratios for GWG Z−scores and Stillbirth from Key Sensitivity Analyses. This table contains adjusted odds ratios for the association between GWG z−scores and stillbirth for key sensitivity analyses, including restricting to stillbirths estimated to have died ≤1 day before delivery and analyzing stillbirths by gestational age at delivery (< 28 vs. ≥28 weeks, < 37 vs. ≥37 weeks). Selected GWG z−scores were compared to a referent z−score of 0. Adjusted models involved control for maternal sociodemographic, behavioral, and pregnancy characteristics. [file 12884_2019_2595_MOESM5_ESM.docx]

**Additional File 5. Adjusted Odds Ratios for GWG Z-scores and Stillbirth from Key Sensitivity Analyses**

| **GWG Z-score^a^** | **Restricting to stillbirths estimated to have died ≤1 day before delivery^b^** | **Restricting to stillbirths estimated to have died >1 day before delivery^b^** | **Restricting to stillbirths alive at last prenatal visit; calculating stillbirths’**  **GWG z-scores using weight/GA at last prenatal visit^b^** | **Excluding pregnancies with macerated stillbirths^b^** | **Restricting to stillbirths delivered <28 weeks^b^** | **Restricting to stillbirths delivered ≥28 weeks^b^** | **Restricting to stillbirths delivered <37 weeks^b^** | **Restricting to stillbirths delivered ≥37 weeks^c^** | **Restricting to non-anomalous antepartum stillbirths or non-anomalous live births^b^** | **Restricting to intrapartum stillbirths^c^** |
| --- | --- | --- | --- | --- | --- | --- | --- | --- | --- | --- |
| **-2.5** | 2.35 (1.64, 3.35) | 2.33 (1.59, 3.42) | 1.69 (1.06, 2.71) | 2.21 (1.63, 3.00) | 2.49 (1.64, 3.80) | 2.22 (1.56, 3.17) | 2.63 (1.90, 3.65) | 1.09 (0.53, 2.26) | 2.46 (1.76, 3.44) | 1.66 (0.80, 3.43) |
| **-2.0** | 1.86 (1.42, 2.44) | 1.89 (1.42, 2.52) | 1.42 (1.00, 2.02) | 1.80 (1.43, 2.26) | 2.00 (1.46, 2.74) | 1.79 (1.37, 2.34) | 2.05 (1.61, 2.62) | 1.06 (0.62, 1.82) | 1.95 (1.52, 2.51) | 1.50 (0.87, 2.59) |
| **-1.5** | 1.48 (1.23, 1.79) | 1.54 (1.27, 1.87) | 1.20 (0.95, 1.52) | 1.46 (1.25, 1.71) | 1.60 (1.29, 1.99) | 1.45 (1.21, 1.74) | 1.60 (1.36, 1.90) | 1.03 (0.72, 1.48) | 1.55 (1.31, 1.84) | 1.36 (0.94, 1.98) |
| **-1.0** | 1.21 (1.07, 1.36) | 1.27 (1.13, 1.43) | 1.04 (0.91, 1.19) | 1.22 (1.11, 1.34) | 1.30 (1.14, 1.49) | 1.20 (1.07, 1.34) | 1.28 (1.16, 1.42) | 1.00 (0.81, 1.24) | 1.26 (1.14, 1.40) | 1.24 (0.98, 1.55) |
| **-0.5** | 1.05 (0.98, 1.12) | 1.09 (1.03, 1.16) | 0.96 (0.91, 1.03) | 1.06 (1.01, 1.12) | 1.11 (1.03, 1.19) | 1.05 (0.98, 1.11) | 1.08 (1.03, 1.14) | 0.99 (0.90, 1.10) | 1.08 (1.02, 1.14) | 1.11 (0.99, 1.26) |
| **0** | 1.00 (1.00, 1.00) | 1.00 (1.00, 1.00) | 1.00 (1.00, 1.00) | 1.00 (1.00, 1.00) | 1.00 (1.00, 1.00) | 1.00 (1.00, 1.00) | 1.00 (1.00, 1.00) | 1.00 (1.00, 1.00) | 1.00 (1.00, 1.00) | 1.00 (1.00, 1.00) |
| **0.5** | 1.07 (0.95, 1.19) | 0.98 (0.89, 1.08) | 1.17 (1.08, 1.27) | 1.03 (0.95, 1.11) | 0.97 (0.87, 1.09) | 1.06 (0.96, 1.17) | 1.02 (0.94, 1.11) | 1.03 (0.89, 1.19) | 1.02 (0.93, 1.11) | 0.89 (0.73, 1.08) |
| **1.0** | 1.22 (0.95, 1.58) | 1.01 (0.81, 1.27) | 1.50 (1.24, 1.81) | 1.12 (0.93, 1.35) | 0.99 (0.76, 1.29) | 1.20 (0.96, 1.50) | 1.11 (0.91, 1.36) | 1.08 (0.77, 1.51) | 1.10 (0.89, 1.35) | 0.79 (0.50, 1.25) |
| **1.5** | 1.45 (0.95, 2.20) | 1.07 (0.74, 1.54) | 1.98 (1.45, 2.70) | 1.25 (0.93, 1.69) | 1.03 (0.67, 1.58) | 1.40 (0.97, 2.01) | 1.25 (0.90, 1.73) | 1.13 (0.65, 1.97) | 1.22 (0.87, 1.71) | 0.70 (0.33, 1.48) |
| **2.0** | 1.71 (0.96, 3.07) | 1.12 (0.67, 1.87) | 2.62 (1.70, 4.04) | 1.40 (0.92, 2.12) | 1.07 (0.59, 1.95) | 1.63 (0.98, 2.71) | 1.40 (0.89, 2.20) | 1.19 (0.55, 2.59) | 1.35 (0.85, 2.16) | 0.62 (0.22, 1.76) |
| **2.5** | 2.03 (0.96, 4.27) | 1.18 (0.61, 2.28) | 3.47 (1.99, 6.04) | 1.57 (0.92, 2.67) | 1.11 (0.51, 2.41) | 1.91 (1.00, 3.64) | 1.57 (0.88, 2.81) | 1.26 (0.46, 3.40) | 1.50 (0.82, 2.74) | 0.55 (0.14, 2.09) |

^a^Selected GWG z−scores were compared to a referent z−score of 0. Among women with singleton pregnancies, GWG z−scores of −2.5, −2.0, −1.5, −1.0, −0.5, 0, 0.5, 1.0, 1.5, 2.0, and 2.5 correspond to the following 40−week total GWG: in women with pre−pregnancy class 1 obesity, −5.0 lb, 0.1 lb, 5.9 lb, 12.4 lb, 19.9 lb, 28.4 lb, 38.1 lb, 49.2 lb, 61.8 lb, 76.2 lb, and 92.5 lb, respectively; in women with pre−pregnancy class 2 obesity, −13.8 lb, −8.8 lb, −2.9 lb, 4.1 lb, 12.3 lb, 21.9 lb, 33.2 lb, 46.6 lb, 62.3 lb, 80.7 lb, and 102.4 lb, respectively; and among women with pre−pregnancy class 3 obesity, −22.7 lb, −18.0 lb, −12.2 lb, −5.0 lb, 4.0 lb, 15.1 lb, 28.9 lb, 46.0 lb, 67.1 lb, 93.4 lb, and 125.9 lb, respectively. Among women with dichorionic/diamniotic twin pregnancies and pre−pregnancy obesity, GWG z−scores of −2.5, −2.0, −1.5, −1.0, −0.5, 0, 0.5, 1.0, 1.5, 2.0, and 2.5 correspond to a 38−week total GWG of −2.1 lb, 2.7 lb, 8.4 lb, 15.4 lb, 23.8 lb, 33.9 lb, 46.2 lb, 60.9 lb, 78.8 lb, 100.3 lb, and 126.3 lb, respectively.

^b^Adjusted for maternal age at delivery, maternal race and ethnicity, study site, maternal education, marital status/cohabitating, health insurance type, trimester prenatal care began, family income in the last 12 months, WIC enrollment, smoking or alcohol consumption during the 3 months prior to pregnancy, lifetime drug use, pregnancy history, history of hypertension, history of preexisting diabetes, and history of thyroid disorder.

^c^Adjusted for maternal age at delivery, maternal race and ethnicity, maternal education, marital status/cohabitating, health insurance type, trimester prenatal care began, family income in the last 12 months, WIC enrollment, smoking or alcohol consumption during the 3 months prior to pregnancy, pregnancy history, history of hypertension, and history of preexisting diabetes. Generalized estimating equations could not be used for this model due to inadequate sample size.
