# Supplementary material for: The association between gestational weight gain z-score and stillbirth: a case-control study
Source: BMC Pregnancy Childbirth. 2019 Nov 29;19:451. doi: 10.1186/s12884-019-2595-x (PMC6883690; doi:10.1186/s12884-019-2595-x)
Supplement: Supplementary file 6 — Additional file 6. Adjusted Odds Ratios for GWG Z−scores and Stillbirth from Additional Sensitivity Analyses. This table contains adjusted odds ratios for the association between GWG z−scores and stillbirth for various additional sensitivity analyses (e.g., excluding women with a GA at delivery that exceeded the limit on the GWG z−score charts; using weight at last prenatal visit for women missing delivery weight). Selected GWG z−scores were compared to a referent z−score of 0. Adjusted models involved control for maternal sociodemographic, behavioral, and pregnancy characteristics. [file 12884_2019_2595_MOESM6_ESM.docx]

**Additional File 6. Adjusted Odds Ratios for GWG Z-scores and Stillbirth from Additional Sensitivity Analyses**

| **GWG**  **Z-score^a^** | **Excluding probable COD due to hematologic conditions, fetal abnormalities^b^** | **Excluding possible or probable COD due to hematologic conditions, fetal abnormalities^b^** | **Excluding present, possible, or probable COD due to hematologic conditions, fetal abnormalities^b^** | **Excluding stillbirths with estimated GA at death <20 weeks^b^** | **Excluding women with a GA at delivery that exceeded the limit on the GWG z-score charts^b^** | **Using weight at last prenatal visit for women missing delivery weight^b^** | **Controlling for weight and height squared as separate variables^b^** |
| --- | --- | --- | --- | --- | --- | --- | --- |
| **-2.5** | 2.53 (1.77, 3.62) | 2.46 (1.72, 3.51) | 2.29 (1.56, 3.35) | 2.37 (1.75, 3.22) | 2.33 (1.69, 3.21) | 2.19 (1.64, 2.91) | 2.28 (1.69, 3.08) |
| **-2.0** | 2.01 (1.54, 2.62) | 1.96 (1.50, 2.55) | 1.86 (1.39, 2.47) | 1.90 (1.51, 2.38) | 1.88 (1.48, 2.39) | 1.79 (1.44, 2.21) | 1.84 (1.47, 2.31) |
| **-1.5** | 1.59 (1.33, 1.91) | 1.56 (1.30, 1.87) | 1.51 (1.24, 1.83) | 1.52 (1.30, 1.78) | 1.52 (1.29, 1.79) | 1.46 (1.26, 1.69) | 1.49 (1.28, 1.74) |
| **-1.0** | 1.29 (1.15, 1.44) | 1.27 (1.14, 1.42) | 1.25 (1.11, 1.41) | 1.24 (1.13, 1.37) | 1.25 (1.13, 1.38) | 1.22 (1.12, 1.34) | 1.23 (1.12, 1.35) |
| **-0.5** | 1.09 (1.03, 1.16) | 1.08 (1.02, 1.15) | 1.08 (1.01, 1.15) | 1.07 (1.02, 1.13) | 1.08 (1.02, 1.13) | 1.07 (1.02, 1.12) | 1.07 (1.01, 1.12) |
| **0** | 1.00 (1.00, 1.00) | 1.00 (1.00, 1.00) | 1.00 (1.00, 1.00) | 1.00 (1.00, 1.00) | 1.00 (1.00, 1.00) | 1.00 (1.00, 1.00) | 1.00 (1.00, 1.00) |
| **0.5** | 1.00 (0.90, 1.10) | 1.01 (0.92, 1.11) | 1.00 (0.91, 1.11) | 1.03 (0.95, 1.11) | 1.01 (0.93, 1.09) | 1.02 (0.94, 1.10) | 1.03 (0.95, 1.11) |
| **1.0** | 1.05 (0.84, 1.31) | 1.08 (0.86, 1.34) | 1.06 (0.84, 1.34) | 1.12 (0.93, 1.34) | 1.08 (0.90, 1.30) | 1.09 (0.91, 1.30) | 1.12 (0.93, 1.34) |
| **1.5** | 1.13 (0.78, 1.63) | 1.18 (0.82, 1.69) | 1.15 (0.78, 1.68) | 1.25 (0.93, 1.69) | 1.18 (0.88, 1.59) | 1.19 (0.89, 1.59) | 1.25 (0.93, 1.68) |
| **2.0** | 1.22 (0.73, 2.03) | 1.29 (0.78, 2.13) | 1.24 (0.73, 2.11) | 1.40 (0.92, 2.13) | 1.30 (0.86, 1.96) | 1.31 (0.88, 1.94) | 1.39 (0.92, 2.11) |
| **2.5** | 1.32 (0.69, 2.53) | 1.41 (0.74, 2.69) | 1.35 (0.68, 2.66) | 1.57 (0.92, 2.68) | 1.42 (0.83, 2.41) | 1.43 (0.86, 2.38) | 1.56 (0.91, 2.65) |

^a^Selected GWG z−scores were compared to a referent z−score of 0. Among women with singleton pregnancies, GWG z−scores of −2.5, −2.0, −1.5, −1.0, −0.5, 0, 0.5, 1.0, 1.5, 2.0, and 2.5 correspond to the following 40−week total GWG: in women with pre−pregnancy class 1 obesity, −5.0 lb, 0.1 lb, 5.9 lb, 12.4 lb, 19.9 lb, 28.4 lb, 38.1 lb, 49.2 lb, 61.8 lb, 76.2 lb, and 92.5 lb, respectively; in women with pre−pregnancy class 2 obesity, −13.8 lb, −8.8 lb, −2.9 lb, 4.1 lb, 12.3 lb, 21.9 lb, 33.2 lb, 46.6 lb, 62.3 lb, 80.7 lb, and 102.4 lb, respectively; and among women with pre−pregnancy class 3 obesity, −22.7 lb, −18.0 lb, −12.2 lb, −5.0 lb, 4.0 lb, 15.1 lb, 28.9 lb, 46.0 lb, 67.1 lb, 93.4 lb, and 125.9 lb, respectively. Among women with dichorionic/diamniotic twin pregnancies and pre−pregnancy obesity, GWG z−scores of −2.5, −2.0, −1.5, −1.0, −0.5, 0, 0.5, 1.0, 1.5, 2.0, and 2.5 correspond to a 38−week total GWG of −2.1 lb, 2.7 lb, 8.4 lb, 15.4 lb, 23.8 lb, 33.9 lb, 46.2 lb, 60.9 lb, 78.8 lb, 100.3 lb, and 126.3 lb, respectively.

^b^COD, Cause of Death. Models were adjusted for maternal age at delivery, maternal race and ethnicity, study site, maternal education, marital status/cohabitating, health insurance type, trimester prenatal care began, family income in the last 12 months, WIC enrollment, smoking or alcohol consumption during the 3 months prior to pregnancy, lifetime drug use, pregnancy history, history of hypertension, history of preexisting diabetes, and history of thyroid disorder.
