# Supplementary material for: The association between gestational weight gain z-score and stillbirth: a case-control study
Source: BMC Pregnancy Childbirth. 2019 Nov 29;19:451. doi: 10.1186/s12884-019-2595-x (PMC6883690; doi:10.1186/s12884-019-2595-x)
Supplement: Supplementary file 7 — Additional file 7. Adjusted Odds Ratios for GWG Z−scores and Stillbirth by Cause-of-Death Groupings. This table contains adjusted odds ratios for the association between GWG z−scores and stillbirth by cause-of-death groupings [23]. The first sensitivity analysis was restricted to stillbirths with a probable cause of death, possible cause of death, or present condition related to placental abnormalities. The second was restricted to stillbirths with a probable cause of death, possible cause of death, or present condition related to maternal medical conditions excluding hypertension [23]. The third was restricted to stillbirths with a probable cause of death, possible cause of death, or present condition related to obstetric conditions [23]. Selected GWG z−scores were compared to a referent z−score of 0. Adjusted models involved control for maternal sociodemographic, behavioral, and pregnancy characteristics. [file 12884_2019_2595_MOESM7_ESM.docx]

| **GWG Z-score^a^** | **Restricted to stillbirths with placental abnormalities^b,c^** | **Restricted to stillbirths with maternal medical conditions, excluding hypertension^b,d^** | **Restricted to stillbirths with obstetric complications of pregnancy^b,e^** |
| --- | --- | --- | --- |
| **-2.5** | 2.61 (1.66, 4.09) | 2.10 (1.30, 3.40) | 2.66 (1.72, 4.12) |
| **-2.0** | 2.06 (1.47, 2.88) | 1.76 (1.23, 2.53) | 2.02 (1.45, 2.81) |
| **-1.5** | 1.63 (1.29, 2.05) | 1.48 (1.15, 1.89) | 1.53 (1.22, 1.93) |
| **-1.0** | 1.31 (1.14, 1.51) | 1.25 (1.07, 1.47) | 1.20 (1.04, 1.39) |
| **-0.5** | 1.10 (1.03, 1.19) | 1.10 (1.01, 1.19) | 1.03 (0.95, 1.11) |
| **0** | 1.00 (1.00, 1.00) | 1.00 (1.00, 1.00) | 1.00 (1.00, 1.00) |
| **0.5** | 0.98 (0.88, 1.10) | 0.96 (0.84, 1.10) | 1.13 (1.00, 1.27) |
| **1.0** | 1.02 (0.79, 1.33) | 0.95 (0.70, 1.31) | 1.40 (1.07, 1.84) |
| **1.5** | 1.09 (0.71, 1.68) | 0.96 (0.58, 1.60) | 1.82 (1.17, 2.82) |
| **2.0** | 1.16 (0.64, 2.12) | 0.97 (0.48, 1.97) | 2.36 (1.28, 4.35) |
| **2.5** | 1.24 (0.57, 2.68) | 0.98 (0.39, 2.43) | 3.07 (1.40, 6.71) |

**Additional File 7. Adjusted Odds Ratios for GWG Z-scores and Stillbirth by Cause-of-Death Groupings**

^a^Selected GWG z−scores were compared to a referent z−score of 0. Among women with singleton pregnancies, GWG z−scores of −2.5, −2.0, −1.5, −1.0, −0.5, 0, 0.5, 1.0, 1.5, 2.0, and 2.5 correspond to the following 40−week total GWG: in women with pre−pregnancy class 1 obesity, −5.0 lb, 0.1 lb, 5.9 lb, 12.4 lb, 19.9 lb, 28.4 lb, 38.1 lb, 49.2 lb, 61.8 lb, 76.2 lb, and 92.5 lb, respectively; in women with pre−pregnancy class 2 obesity, −13.8 lb, −8.8 lb, −2.9 lb, 4.1 lb, 12.3 lb, 21.9 lb, 33.2 lb, 46.6 lb, 62.3 lb, 80.7 lb, and 102.4 lb, respectively; and among women with pre−pregnancy class 3 obesity, −22.7 lb, −18.0 lb, −12.2 lb, −5.0 lb, 4.0 lb, 15.1 lb, 28.9 lb, 46.0 lb, 67.1 lb, 93.4 lb, and 125.9 lb, respectively. Among women with dichorionic/diamniotic twin pregnancies and pre−pregnancy obesity, GWG z−scores of −2.5, −2.0, −1.5, −1.0, −0.5, 0, 0.5, 1.0, 1.5, 2.0, and 2.5 correspond to a 38−week total GWG of −2.1 lb, 2.7 lb, 8.4 lb, 15.4 lb, 23.8 lb, 33.9 lb, 46.2 lb, 60.9 lb, 78.8 lb, 100.3 lb, and 126.3 lb, respectively.

^b^Adjusted for maternal age at delivery, maternal race and ethnicity, study site, maternal education, marital status/cohabitating, health insurance type, trimester prenatal care began, family income in the last 12 months, WIC enrollment, smoking or alcohol consumption during the 3 months prior to pregnancy, lifetime drug use, pregnancy history, history of hypertension, history of preexisting diabetes, and history of thyroid disorder.

^c^Restricted to stillbirths with a probable cause of death, possible cause of death, or present condition related to placental abnormalities (e.g., uteroplacental insufficiency, maternal vascular disorders, etc.).

^d^Restricted to stillbirths with a probable cause of death, possible cause of death, or present condition related to maternal medical conditions, excluding hypertension (e.g., diabetes, antiphospholipid syndrome, thyroid disorder, etc.).

^e^Restricted to stillbirths with a probable cause of death, possible cause of death, or present condition related to obstetric conditions (e.g., placental abruption; complications of multiple gestations; the combination of preterm labor, preterm premature rupture of membranes, and cervical insufficiency, etc.).
